# Real-World Data on Combined EGFR-TKI and Crizotinib Treatment for Acquired and De Novo *MET* Amplification in Patients with Metastatic *EGFR*-Mutated NSCLC

**DOI:** 10.3390/ijms241713077

**Published:** 2023-08-23

**Authors:** Edyta M. Urbanska, Morten Grauslund, Peter R. Koffeldt, Sarah L. B. Truelsen, Johan O. Löfgren, Junia C. Costa, Linea C. Melchior, Jens B. Sørensen, Eric Santoni-Rugiu

**Affiliations:** 1 Department of Oncology, Rigshospitalet, Copenhagen University Hospital, DK-2100 Copenhagen, Denmark; jens.benn.soerensen@regionh.dk; 2 Department of Pathology, Rigshospitalet, Copenhagen University Hospital, DK-2100 Copenhagen, Denmark; morten.grauslund@regionh.dk (M.G.); peter.rindom.koffeldt@regionh.dk (P.R.K.); sarah.truelsen@regionh.dk (S.L.B.T.); linea.cecilie.melchior@regionh.dk (L.C.M.); 3 Department of Clinical Physiology and Nuclear Medicine, Rigshospitalet, Copenhagen University Hospital, DK-2100 Copenhagen, Denmark; johan.olof.loefgren@regionh.dk; 4 Department of Radiology, Rigshospitalet, Copenhagen University Hospital, DK-2100 Copenhagen, Denmark; junia.cardoso.costa@regionh.dk; 5 Department of Clinical Medicine, University of Copenhagen, DK-2200 Copenhagen, Denmark

**Keywords:** *EGFR*-mutated NSCLC, EGFR-TKI, acquired *MET* amplification, de novo *MET* amplification, Crizotinib, combined targeted therapy

## Abstract

Amplification of the *mesenchymal epithelial transition* (*MET*) gene is a mechanism of acquired resistance to epidermal growth factor receptor (EGFR)-tyrosine-kinase-inhibitors (TKIs) in over 20% of patients with advanced *EGFR*-mutated (*EGFR*m+) non-small lung cancer (NSCLC). However, it may also occur de novo in 2–8% of *EGFR*m+ NSCLC cases as a potential mechanism of intrinsic resistance. These patients represent a group with unmet needs, since there is no standard therapy currently approved. Several new MET inhibitors are being investigated in clinical trials, but the results are awaited. Meanwhile, as an alternative strategy, combinations of EGFR-TKIs with the MET/ALK/ROS1-TKI Crizotinib may be used in this setting, despite this use is principally off-label. Thus, we studied five of these *MET* amplified cases receiving EGFR-TKI and Crizotinib doublet after progression on EGFR-TKI treatment to assess the benefits and challenges related to this combination and the possible occurrence of genomic and phenotypic co-alterations. Furthermore, we compared our cases with other real-world reports on Crizotinib/EGFR-TKI combinations, which appeared effective, especially in patients with high-level *MET* amplification. Yet, we observed that the co-occurrence of other genomic and phenotypical alterations may affect the response to combined EGFR-TKI and Crizotinib. Finally, given the heterogeneity of *MET* amplification, the diagnostic methods for assessing it may be discrepant. In this respect, we observed that for optimal detection, immunohistochemistry, fluorescence in situ hybridization, and next-generation sequencing should be used together, as these methods possess different sensitivities and complement each other in characterizing *MET* amplification. Additionally, we addressed the issue of managing *EGFR*-mutated NSCLC patients with de novo *MET* amplification causing primary EGFR-TKI resistance. We conclude that, while data from clinical trials with new MET inhibitors are still pending, adding Crizotinib to EGFR-TKI in NSCLC patients acquiring *MET* amplification at progression on EGFR-TKI monotherapy is a reasonable approach, with a progression-free survival of 3–19 months.

## 1. Introduction

For patients with *EGFR*-mutated (*EGFR*m+) NSCLC treated with EGFR-TKIs, there is no specific recommendation at the time of progression. Several clinical practices, including chemotherapy alone or in combination with an angiogenesis inhibitor (Bevacizumab) and single-agent immunotherapy or in combination with chemotherapy, result in a low objective response rate (ORR) and a short progression-free survival (PFS) [1,2,3,4]. Therefore, a better understanding of the reasons for treatment failure using the detection mechanism of acquired and/or intrinsic resistance in tumor rebiopsies and/or circulating free DNA (cfDNA) is crucial for defining further therapeutic options in terms of precision medicine [5]. 

In *EGFR*m+ NSCLC, MET signaling deregulation due to *MET* gene amplification is the most frequent off-target mechanism of acquired resistance during first-line treatment with Osimertinib. It occurs with a frequency ranging between 7% and 20% of cases in different studies with various numbers of investigated patients, types of specimens, and utilized methods for *MET* amplification detection [6,7,8,9]. However, in a recent study, six out of nine (66%) patients receiving first-line Osimertinib were reported to develop subclonal and heterogeneous *MET* amplification during treatment [10], which might suggest that the frequency of acquired *MET* amplification during first-line Osimertinib treatment is even higher than otherwise reported. In any case, comparable frequencies of acquired *MET* amplification have been observed after treatment with first- and second-generation EGFR-TKIs and during second- or next-line treatment with Osimertinib [7,8,9]. There are currently several promising MET-TKIs, bispecific antibodies targeting EGFR and MET, and MET-targeting antibody-drug conjugates (ADCs) being investigated in clinical trials for NSCLC patients with MET-deregulation [11,12]. Two MET-specific TKIs, Capmatinib and Tepotinib, are already FDA and EMA approved for NSCLC patients with *MET* exon 14 skipping mutations (*MET*ex14) [11,12], but not for *MET* amplification. The FDA, on the basis of results from the PROFILE 1001 trial, has also granted Crizotinib, a type 1a MET-TKI, which also inhibits the ROS1 and ALK kinases, a breakthrough therapy designation for the treatment of patients with metastatic NSCLC with *MET*ex14 alterations progressing after platinum-based chemotherapy [13].

Initial phase II trials showed an objective response rate (ORR) of *MET*-amplified NSCLC to Crizotinib of approximately 30% and limited median PFS (max 5 months) and overall survival (OS) (max 7.7 months) [14,15]. Yet new data show that Crizotinib-treated NSCLC patients with wildtype (wt) *EGFR* and high-level *MET* amplification may achieve a median OS of >11 months and that Crizotinib is more effective than chemotherapy or immunotherapy as first-line therapy in this setting [16,17]. In this regard, Crizotinib exhibited more efficacy in NSCLC patients with wt *EGFR* and *MET*ex14 with a median OS of 22.8 months than in those with *MET* amplification (median OS 5.4 months) [18]. *MET* amplification as a driver can also be found in patients with squamous or sarcomatoid phenotypes of NSCLC, in whom response to Crizotinib may be poorer [19,20]. Furthermore, results from a small retrospective study showed activity of Crizotinib as monotherapy in *MET* amplified patients progressing on first-line platin-based chemotherapy [21].

Given the availability of the above-mentioned anti-MET drugs and their proven effect in preclinical models and phase I–II trials, *MET* amplification as a mechanism of resistance to EGFR-TKIs is clinically actionable in a combination approach attempting concomitant inhibition of EGFR and MET signaling [7,11,12,22,23,24,25]. However, until now, none of the available anti-MET drugs have been approved in this setting [26]. Preclinical data from *EGFR* T790M-negative cell lines with acquired Osimertinib resistance due to *MET* amplification indicated that Osimertinib combined with Crizotinib or other investigational MET-TKIs may circumvent resistance to EGFR-TKIs in vivo and in vitro [27]. Despite that newer MET-TKIs such as Capmatinib, Savolitinib, Tepotinib, and Cabozantinib have been investigated in *EGFR*m+ NSCLC patients with acquired *MET* amplification [22,28,29,30], Crizotinib remains the most available therapeutic option for these patients.

We present five different cases of *EGFR*m+ NSCLC patients with acquired *MET* amplification as a resistance mechanism at the first, second, or later progression on EGFR-TKIs, who were treated by combining these drugs with Crizotinib. The cases also serve to illustrate that because of tumor heterogeneity, only certain tumor clones may harbor *MET* amplification in NSCLC, thereby making their detection challenging in small tumor biopsies [11,31,32]. Comparing our observations with the relatively limited number of reported real-world cases, we discuss the clinical impact of *MET* amplification in the context of different co-existing genomic alterations, challenging diagnostic methods, and efficacy of combined treatment with EGFR-TKIs and Crizotinib. 

## 2. Results

Five real-world cases are presented, reflecting the heterogeneous configuration of *EGFR*m+ NSCLC acquiring *MET* amplification during EGFR-TKI treatment. In all these cases a combination of EGFR-TKI and Crizotinib was administered in different treatment lines and with variable outcomes. Furthermore, these cases illustrate the challenges in the diagnostics of *MET* amplification (including discrepancies between IHC or FISH and NGS).

### 2.1. Case 1: Metastatic NSCLC with EGFR ex19del and Acquired MET Amplification: Short-Term Complete Response (CR) by Combining Crizotinib with Osimertinib

**Figure 1:**
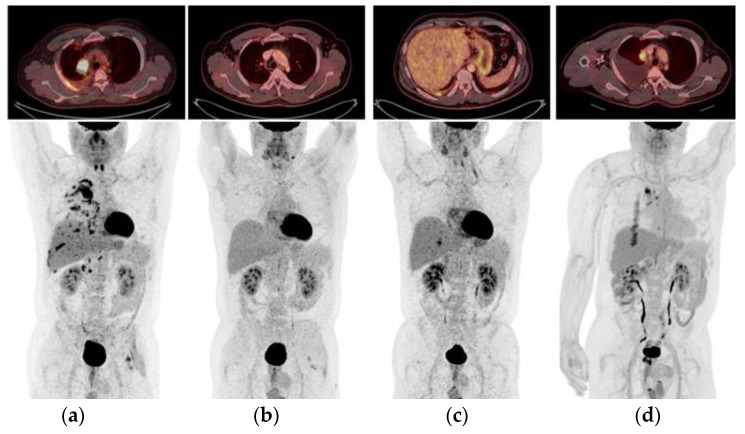
The pictures present four assessments performed during treatment of case 1. (**a**) Baseline positron-emission tomography (PET)/computed tomography (CT): A 5 cm 18F-Fluoro-deoxy-glucose (FDG)-avid tumor in the pulmonary upper right lobe and extensive metastatic spread including multiple small lesions in both lungs, multiple right-sided pleural lesions, mediastinal and right hilar lymph nodes, and several retroperitoneal lymph nodes. (**b**) After 6 months: PET/CT shows complete metabolic and considerable structural regression of all prior lesions. (**c**) At 9 months, control PET/CT shows, posteriorly, on the right side, an intense FDG uptake in relation to the pleura and pleural effusion, without any evident tumor seen on CT. The finding was suspicious for relapse. (**d**) Metabolic and structural progression of primary tumor in the right lung. Malignant mediastinal and right hilar lymph nodes. Increased metabolic activity posteriorly in the increasing right-sided pleural effusion—suspicion of malignant pleural effusion.

A 53-year-old, North-African male, never-smoker, with a performance status of (PS) 1, was diagnosed by tissue biopsy with T4N2M1a lung adenocarcinoma (LAC) (Figure 1a) harboring *EGFR* ex19del (p.E746_A750del). This variant was also detected in baseline plasma cfDNA, with a variant allele frequency (VAF) of 0.33%. The patient achieved a complete objective response (OR), as evaluated by positron-emission tomography (PET)/computed tomography (CT) (Figure 1b), and showed disappearance of the *EGFR* ex19del in plasma during first-line Osimertinib treatment. However, he progressed after 9 months with a re-occurrence of *EGFR* ex19del in cfDNA (VAF 0.29%), and ^18^F-Fluoro-deoxy-glucose (FDG)-PET/CT revealed progressing lesions in the right pleura and pancreas (Figure 1c). Rebiopsy from the right pleura showed multiple potential resistance mechanisms. IHC was “MET-positive” showing overexpression (3+) of MET receptor in 70% of tumor cells, whereas FISH analysis revealed clones of tumor cells with *MET* amplification (53% of tumor cells with ≥5 *MET* copies, average *MET*-GCN/cell of 8.2, and 10% of tumor cells with gene clusters of >15 copies). NGS analysis detected, in addition to the founder *EGFR* ex19del, mutations of *TP53* (p.Q331*), *FGFR2* (p.R201L), and *SMAD4* (p.L540R) together with *JAK3* amplification (7 copies). However, *MET* amplification was not captured by NGS, possibly reflecting the above-mentioned clonal heterogeneity of *MET* amplification. 

The patient started a standard dose of Crizotinib (250 mg BD) and continued Osimertinib 80 mg QD. The treatment was feasible, and only manageable diarrhea grade 1–2 was observed. 

**Figure 2:**
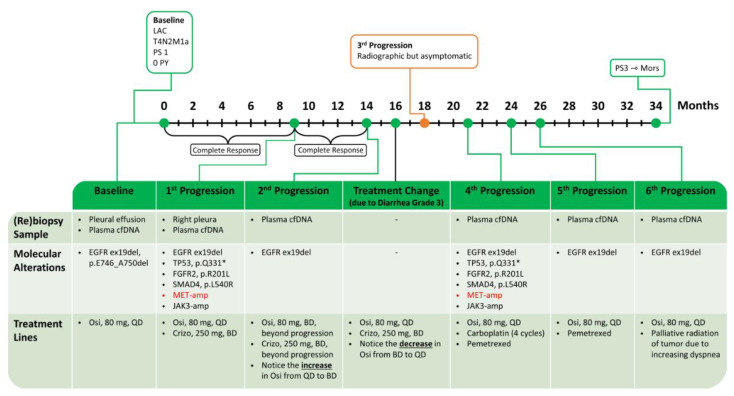
Summary of treatment for case 1. *MET* alterations in red. LAC: lung adenocarcinoma; PS: performance status; PY: pack-years; cfDNA: cell-free DNA; Osi: Osimertinib; Crizo: Crizotinib.

The patient was treated for 5 months, achieving complete clinical and radiographic response, despite the significant genomic co-alterations. However, a cfDNA control sample showed persistent *EGFR* ex19del with a VAF increasing to 3.68%, a finding that can predict worse survival and impending progression [33]. Accordingly, the patient was offered to escalate the dose of Osimertinib (80 mg BD) while continuing Crizotinib, but that was feasible for only two months due to toxicity (grade 3 diarrhea), which necessitated resuming the initial Osimertinib dose. The liquid biopsies taken every second month continuously revealed the persistence of the *EGFR* ex19del in the cfDNA. Next FDG-PET/CT performed nine months after combination treatment showed intrathoracic progression (Figure 1d), but the patient was asymptomatic and continued the treatment beyond progression during the following three months, after which further intrathoracic progression was observed. Examination of the malignant pleural effusion showed the previous mutational profile, i.e., *EGFR* ex19del, *MET* amplification (76% of tumor cells with >6 copies, average *MET*-GCN/cell of 10.3, >10% with *MET* gene clusters), *JAK3* amplification, and *TP53*, *FGFR2*, and *SMAD4* mutation. All these co-alterations could represent mechanisms of resistance to the Osimertinib–Crizotinib combination, yet they were not obvious druggable targets. Thus, Crizotinib was discontinued after 12 months, and the patient began chemotherapy with Carboplatin and Pemetrexed, while continuing Osimertinib. After four cycles, clinical and radiographic regression was observed, but the newly sampled cfDNA displayed persistence of *EGFR* ex19del at a low VAF (0.28%). The patient continued maintenance Pemetrexed together with Osimertinib; however, further progression of the T-site and remaining circulating *EGFR* ex19del (AF 0.18%) were observed after two months. Due to increasing dyspnea, the patient received palliative radiation against the progressing lung tumor (3 Gy × 10 fr) and, given the persistent and increasing circulating *EGFR* ex19del (VAF 1.7%), he continued Osimertinib. The patient’s condition deteriorated quickly, and he died after having reached an OS of 34 months. A schematic time course of this case is shown in Figure 2.

### 2.2. Case 2: Transient Efficacy of Crizotinib for Acquired MET Dysregulations Accompanied by Squamous Cell Transformation

**Figure 3:**
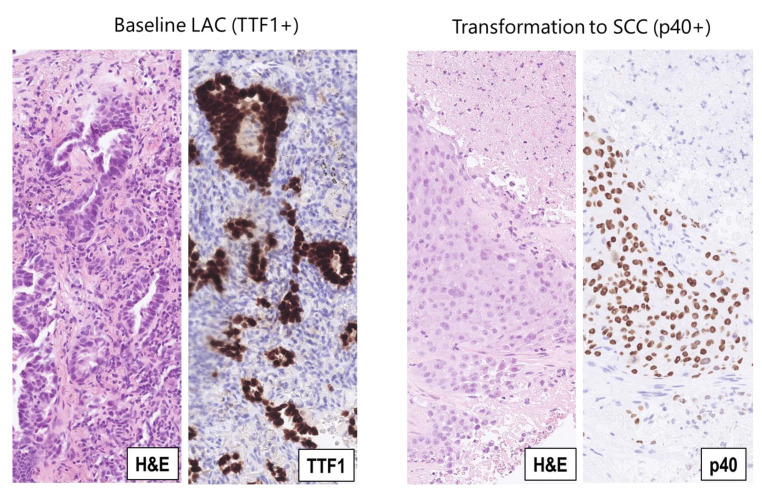
NSCLC histological phenotypes in case 3. **Left panels**: Diagnostic biopsy (baseline) from a metastasis to the thoracic lymph nodal station 4R with phenotype of lung adenocarcinoma (LAC); hematoxylin-eosin staining (H&E) shows malignant adeno-papillary structures, which are positive for the immunohistochemical LAC biomarker TTF1 (TTF1). **Right panels**: Rebiopsy from progressive metastasis in the left lung shows tumor transformation to squamous cell carcinoma (SCC); hematoxylin-eosin staining (H&E) shows a solid, slightly keratinizing tumor tissue, which is positive for the immunohistochemical SCC biomarker p40 (p40). (All magnifications, ×200).

A 70-year-old, Vietnamese, male, smoker with 13 pack-years (py), in PS 1, was diagnosed with T3N2M1c NSCLC, adenocarcinoma type, harboring *EGFR* ex19del (p.E746_T751delinsVA). The patient’s NSCLC progressed after six months of successful treatment with Osimertinib. Analysis of plasma cfDNA did not display any variants among the investigated genes. Rebiopsy from progressive metastasis in the left lower lung lobe showed histology and immune profile of squamous cell carcinoma (SCC) (Figure 3), which suggested that the original NSCLC might have been adeno-squamous and now the squamous component was predominant in this metastasis or that the LAC had transformed to SCC as a phenotypic mechanism of TKI resistance [6,34].

**Figure 4:**
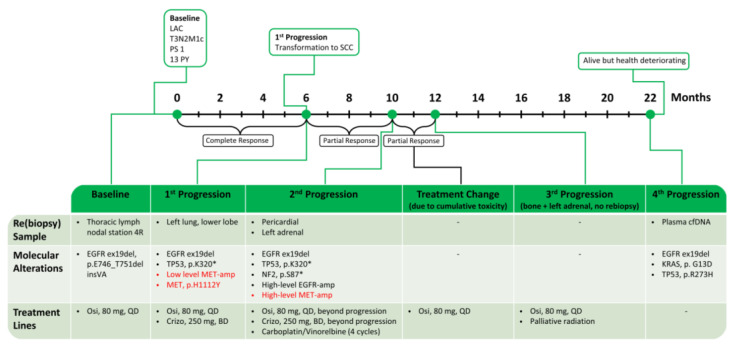
Summary of treatment for case 2. *MET* alterations in red. LAC: lung adenocarcinoma; PS: performance status; PY: pack-years; SCC: squamous cell carcinoma; cfDNA: cell-free DNA; Osi: Osimertinib; and Crizo: Crizotinib.

We identified additional potential mechanisms of acquired Osimertinib resistance in the tumor rebiopsy, such as the *MET* p.H1112Y variant, MET receptor upregulation (MET-IHC 3+ in 70% and 2+ in 30% of tumor cells), and low-level *MET* amplification (61% of tumor cells with ≥4 *MET* copies by FISH analysis) as well as *TP53* mutation (p.K320*). No *MET* amplification was observed in the NGS analysis. The patient was initiated on Crizotinib 250 mg BD, while continuing Osimertinib 80 mg QD. Significant clinical improvement was quickly obtained, and no adverse events were observed. After four months of this combination treatment, growth of a pericardial and a left adrenal metastasis was observed, while other lesions were stable. A second rebiopsy from the left adrenal gland displayed the original founder *EGFR* ex19del and the *TP53* mutation (p.K320*) as well as three acquired potential mechanisms of TKI-resistance mechanisms: mutation of the *NF2* tumor-suppressor gene (p.S87*) and high-level amplification of *EGFR* and *MET* genes (both >10 copies by NGS and 100% of tumor cells with *MET* gene “clusters” of >15 copies as assessed by FISH and resulting in MET-IHC 3+ in 100% of tumor cells). Therefore, the patient received four cycles of chemotherapy with Carboplatin/Vinorelbine, while continuing Crizotinib and Osimertinib. Radiographic evaluation showed a partial response (PR); however, due to cumulative toxicity of the two TKIs, the patient continued the treatment with only Osimertinib. After two months, two symptomatic progressive sites (bone and left adrenal gland) were observed, and local radiation therapy was administered with a good palliative effect, while Osimertinib was continued beyond progression, reaching an OS of 22 months. Yet, the original *EGFR* ex19delins together with new mutations in *KRAS* (p.G13D) and *TP53* (p.R273H) were detected in the last cfDNA alongside further deterioration of the patient’s condition. A schematic time course of this case is shown in Figure 4.

### 2.3. Case 3: Crizotinib Rescues the Third Progression on Osimertinib Associated with Complex Resistance Mechanisms

The patient was an 80-year-old female, smoker (53 py), with reduced lung capacity due to chronic obstructive pulmonary disease, and a previous history of breast cancer (right side mastectomy, postoperative radiation therapy, and one-year treatment with Anastrozole discontinued due to side effects three years before NSCLC diagnosis). She had a PS of 2 and was diagnosed with T3bN1M0 LAC harboring *EGFR* p.L858R and *TP53* p.C277F co-mutations. The patient progressed after two months on first-line Osimertinib. Since tissue rebiopsy was unfeasible, plasma cfDNA was analyzed and exhibited no *EGFR* or *TP53* mutations but the presence of a *KRAS* mutation (p.G12R; VAF 0.31%). The patient was unfit for platin-based doublet chemotherapy but was offered Pemetrexed, continuing Osimertinib in standard doses. After three cycles, we observed radiographic tumor regression and disappearance of the *KRAS* variant from the corresponding cfDNA. However, the treatment was temporarily paused because of toxicity. During the treatment break, cfDNA analysis unveiled the reappearance of the original *EGFR* p.L858R (VAF 0.11%) together with the *TP53* p.C277F (VAF 0.20%) and *KRAS* p.G12R (VAF 0.10%) mutations. The patient was re-challenged with Osimertinib and Pemetrexed in reduced doses. Unfortunately, radiographic progression occurred three months later. Despite the absence of circulating *EGFR/KRAS/TP53* mutations, new cfDNA analysis detected the acquired pathogenic gain-of-function *ALK* p.R1275Q mutation (VAF 0.15%) in the ALK-TK domain. This variant is characteristic of neuroblastomas and supposed to be Crizotinib resistant in both neuroblastomas and NSCLC [35,36,37,38]. We assumed that it may also cause Osimertinib resistance and be sensitive to second-generation ALK-TKIs. Thus, the ALK-TKI, Alectinib, was initiated while continuing Osimertinib, both drugs in reduced dose due to the patient’s fragile status. This treatment was well tolerated with no adverse events and with improved quality of life. Three consecutive CT scans of chest/abdomen performed during the following nine months showed stable disease, while no pathogenic variants were detected in plasma cfDNA. Thus, a liquid biopsy-guided approach at progression in elderly patients with reduced PS and reduced tolerability for tumor rebiopsies may offer feasible and effective therapy guidance, as in this case where it disclosed the option of combining ALK- and EGFR-TKI. An effective combination of Osimertinib and Alectinib has been reported in single cases of disseminated *EGFR*m+ NSCLC becoming resistant to Osimertinib through acquired *ALK* fusions.

**Figure 5:**
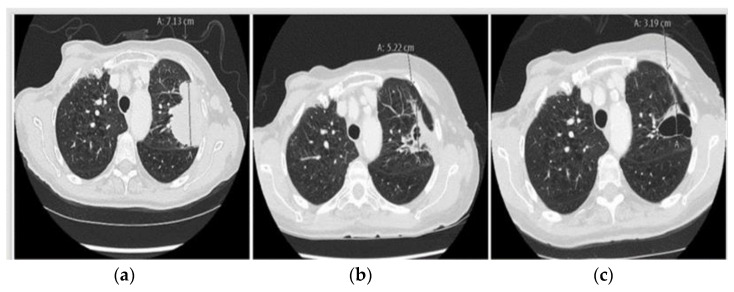
The figure presents three CT scans of the thorax under the treatment course of case 3. “A” represents the lung tumor. (**a**) Tumor progression on Osimertinib–Alectinib. (**b**) Significant tumor regression after three months of combination treatment with Crizotinib and Osimertinib. (**c**) Further tumor regression after six months of treatment with Crizotinib and Osimertinib.

However, to our knowledge, this case is the first to show a durable response to combined Osimertinib–Alectinib treatment when the progression is associated with acquired mutation rather than fusion of *ALK*, indicating that the *ALK* p.R1275Q variant may represent a mechanism of Osimertinib resistance that may be effectively counteracted by Alectinib [39]. Nonetheless, after nine months of PFS, the longest during the entire treatment course, the next progression occurred (Figure 5a). This time a tissue rebiopsy from metastasis in axillar lymph nodes was feasible and revealed acquired *MET* amplification (average *MET*-GCN/cell = 8.3, as assessed by FISH) and overexpression (MET-IHC 3+ in 60% and 2+ in 40% of tumor cells) together with high-level amplification of three other genes (detected by NGS): *PDGFR-A* (26 copies) on chromosome 4, *MYC* (26 copies) on chromosome 8, and *CDK4* (25 copies) on chromosome 12. Furthermore, a new mutation in the tumor suppressor and DNA repair gene *FANCA* (p.Y998*) was observed, together with both the original *EGFR* p.L858R and *TP53* p.C277F co-mutations. Corresponding analysis of cfDNA also identified the *EGFR* p.L858R (VAF 4.1%) and *TP53* p.C277F (VAF 6.8%) variants, but not the *ALK* p.R1275Q variant. The latter was not detected in the rebiopsy either. Thus, the patient discontinued Alectinib and initiated Crizotinib 250 mg QD (due to her fragile status) while continuing Osimertinib 40 mg QD. The first evaluation after three months revealed improvement in symptoms, and CT scan showed reduction of the axillary lymph nodal conglomerate and primary tumor response (Figure 5b). The patient continued this combination treatment for three months displaying further regression without experiencing adverse events (Figure 5c).

**Figure 6:**
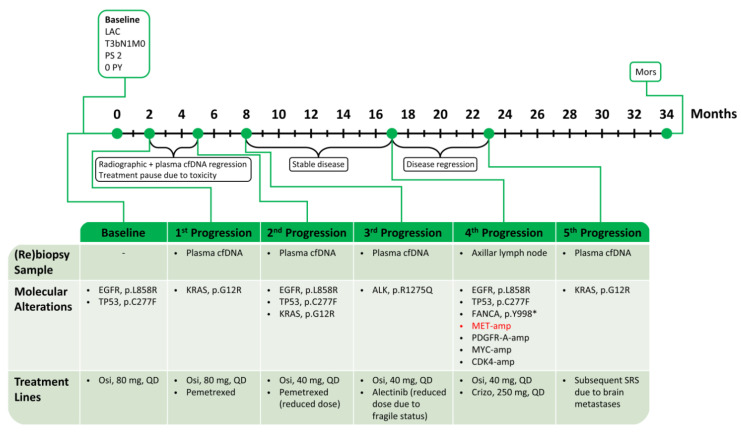
Summary of treatment for case 3. *MET* alterations in red. LAC: lung adenocarcinoma; PS: performance status; PY: pack-years; cfDNA: cell-free DNA; Osi: Osimertinib; Crizo: Crizotinib; and SRS: stereotactic radiosurgery.

Thereafter, new NGS analysis of plasma cfDNA showed reappearance of the circulating *KRAS* p.G12R variant (VAF 0.31%), and the patient progressed intracranially with three brain metastases, which were subsequently treated with stereotactic radiosurgery (SRS). The patient received the fifth line combination treatment for 11 months, after which her condition deteriorated rapidly, and she died shortly thereafter, reaching an OS of 34 months. A schematic time course of this case is shown in Figure 6.

### 2.4. Case 4: Durable Response of Metastatic EGFRm+ NSCLC to Fourth Line Gefitinib Rechallenge Combined with Crizotinib Because of Acquired High-Level MET Amplification

**Figure 7:**
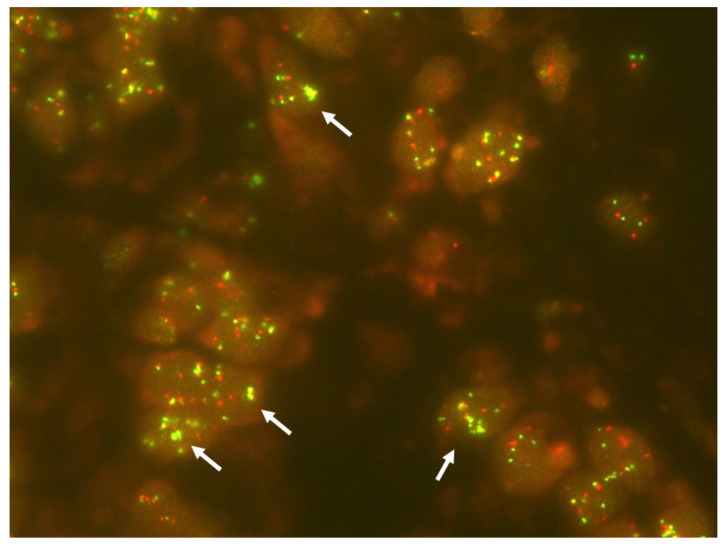
FISH analysis of a rebiopsy from relapsed NSCLC in the right lung at progression on third-line Osimertinib in case 4. High-level *MET* amplification with several tumor cell nuclei exhibiting *MET* gene clusters (arrows), which by convention correspond to >15 copies. (Magnification, ×1000).

An 80-year-old male, in PS 2, former light smoker (2 py), with significant comorbidities (hypertension, diabetes type 2, hypercholesterolemia, and previous apoplexy without sequels), was diagnosed in 2016 with right malignant pleural effusion containing metastatic LAC cells with *EGFR* p.L858R mutation. Following initial Gefitinib (250 mg QD) in first-line treatment (36 months) and Afatinib (20 mg QD) in second-line treatment (five months), supplemented along with palliative radiation therapy against solitary thoracic lesions, the patient received third-line Osimertinib (80 mg QD) and progressed after four months. Rebiopsy from the relapsed tumor in the right pulmonary upper lobe showed an acquired *TP53* mutation (p.L265P) and high-level *MET* amplification (average *MET*-GCN/cell = 9.9 and 15% of tumor cells with *MET* clusters; Figure 7) as well as MET protein upregulation (3+ in 30% and 2+ in 70% of tumor cells) detected by FISH and IHC, respectively, while NGS did not detect the *MET* amplification.

**Figure 8:**
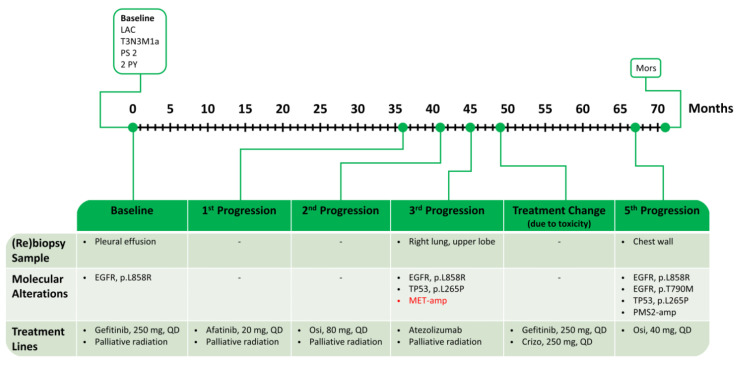
Summary of treatment for case 4. *MET* alterations in red. LAC: lung adenocarcinoma; PS: performance status; PY: pack-years; Osi: Osimertinib; and Crizo: Crizotinib.

Inclusion of the patient in the SAVANNAH trial with the MET-TKI Savolitinib was considered, but the patient declined this possibility. Thus, he was treated with immunotherapy (three cycles of Atezolizumab stretched over four months), resulting in further progression. The combination of EGFR- and MET-TKI was reconsidered. As the patient had experienced gastrointestinal toxicity with Osimertinib, Gefitinib rechallenge together with Crizotinib was initiated. The patient responded for 18 months with an excellent quality of life and no evident adverse events. Thereafter, new progression occurred, and a new rebiopsy from a metastatic lesion in the chest wall revealed, in addition to the founder *EGFR* p.L858R and the previously identified *TP53* p.L265P variants, loss of *MET* amplification and a newly acquired *EGFR* p.T790M mutation, as well as *PMS2* amplification (five copies). Based on these findings, Gefitinib and Crizotinib were discontinued and a re-challenge with Osimertinib in reduced dose of 40 mg QD was initiated. However, the patient’s PS quickly deteriorated, and he deceased after four months. The patient reached an OS of 71 months. A schematic time course of this case is shown in Figure 8.

### 2.5. Case 5: Efficacy of Crizotinib-Osimertinib in EGFRm+ NSCLC Patient Acquiring High-Level MET Amplification after 26 Months of Treatment with Osimertinib

**Figure 9:**
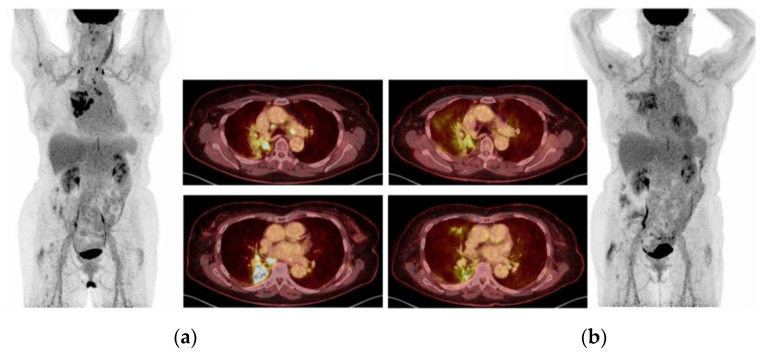
The pictures present the progression after 26 months of Osimertinib treatment in case 5 (**a**) and response to Osimertinib and Crizotinib (**b**). (**a**) Malignant tumor centrally in the right lung. Lymph nodes suspected of malignancy in the right hilus, mediastinum, and at the base of the neck bilaterally. (**b**) Marked regression of metabolic activity in the lung tumor. Complete metabolic remission of previously suspected malignant lymph nodes in the right hilus, mediastinum, and at the base of the neck.

**Figure 10:**
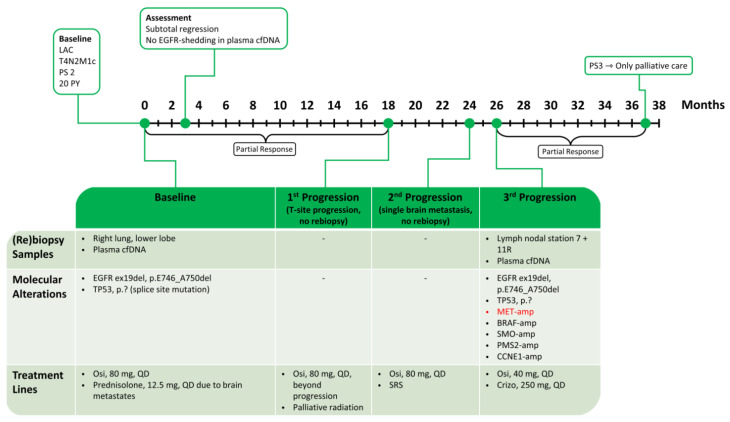
Summary of treatment for case 5. *MET* alterations in red. LAC: lung adenocarcinoma; PS: performance status; PY: pack-years; cfDNA: cell-free DNA; Osi: Osimertinib; Crizo: Crizotinib; and SRS: stereotactic radiosurgery.

A 75-year-old female, smoker (20 py), in a PS of 2, with well-treated hypertension, was diagnosed with LAC in the right lung’s lower lobe with multiple brain and bone metastases and *EGFR* ex19del (p.E746_A750del) and *TP53* (c.673-2A>G, p. potentially affecting the 5’ end splice site of exon 7) co-mutations. A baseline liquid biopsy (plasma cfDNA) showed shedding of the *EGFR* ex19del. The patient was neurologically stable on steroids (Prednisolone 12.5 mg QD) and no additional radiation therapy was needed. She initiated first-line Osimertinib (80 mg QD), and first assessment following three months of treatment showed subtotal regression of cerebral metastases and extracranial PR together with disappearance of the *EGFR* ex19del from the cfDNA. After 18 months of remission, an isolated progression of the T-site was observed, and the patient received palliative local radiation therapy (3 Gy × 10 fractions) while continuing Osimertinib. At the 24-month assessment, growth of a single brain metastasis was observed, and the patient received supplementary SRS radiotherapy (18 Gy × 1 fraction). At the 26-month assessment, oligoprogression was detected intrathoracically (Figure 9a). cfDNA analysis showed the reappearance of the *EGFR* ex19del (VAF 7.82%). The patient continued Osimertinib beyond progression. Rebiopsy from new metastases in thoracic lymph nodal stations 7 and 11R revealed acquired MET overexpression (MET-IHC with 3+ in 80% and 2+ in 20% of tumor cells) associated with high-level *MET* amplification identified by FISH (average *MET*-GCN/cell = 8.04, 77% of tumor cells with ≥6 copies, 15% of tumor cells with clusters), whereas only 5 *MET* gene copies were detected by NGS. This was accompanied by co-amplification of the *BRAF* (five copies), *SMO* (five copies), and *PMS2* (five copies) genes on chromosome 7 (like the *MET* gene), as well as amplification of the *CCNE1* gene (seven copies) on chromosome 19. Furthermore, the original *TP53* mutation was also found, whereas neither phenotypical transformation nor druggable fusions were detected. The patient initiated Crizotinib (250 mg QD) while continuing Osimertinib (40 mg QD), and after three months of this combination treatment, FDG-PET/CT revealed PR (Figure 9b). The treatment was continued without adverse events and with excellent quality of life for a further eight months, after which the patient’s condition deteriorated. The treatment was discontinued due to PS 3, and the patient continued palliative care without further antineoplastic treatment. The patient had hitherto reached an OS of 37 months. A schematic time course of this case is shown in Figure 10.

## 3. Discussion

Despite the advances of the last decade in the treatment of metastatic *EGFR*m+ NSCLC, the 5-year survival remains low and achievable only for ~24% of patients, with a median OS of 36.8 months [40]. *MET* amplification is the most frequent off-target mechanism of acquired resistance to Osimertinib, an EGFR-TKI widely preferred in first-line settings [9,41,42]. Moreover, there is increasing evidence that progression on EGFR-TKI driven by acquired *MET* dysregulation may be further druggable [11,12,43]. This approach requires defining the *MET* gene status in tumor rebiopsies from progressive lesions by thoroughly assessing the possible occurrence of its amplification or mutation as well as the overexpression of the MET receptor protein, since these dysregulations may occur as concurrent subclonal events or in a temporal sequence. Each of these dysregulations—and the extent of their changes—may influence the response to MET targeted therapy. Furthermore, the complexity of acquired resistance to EGFR-TKIs, as also illustrated in the presented cases, with the co-occurrence of different molecular and phenotypical changes, makes it difficult to find a treatment capable of counteracting multiple simultaneous resistance mechanisms. Nevertheless, *MET* amplification is now well proven to function as an oncogenic driver with the ability to significantly impair the response to EGFR-TKIs by causing persistent reactivation of the RAS-RAF-MAPK, PI3K-AKT, and STAT signaling pathways downstream of EGFR [11,12,44]. Thus, targeting both receptors by adding a MET-TKI to EGFR-TKIs is necessary to suppress growth of *EGFR*m+ NSCLCs with co-amplification of the *MET* gene [11,12,44].

However, preliminary results from cell lines suggest that some rare cases of *EGFR*m+ tumors with acquired *MET* amplification may develop dependence on MET activation alone as a result of an entire switch of oncogenic addiction. In such a subset of tumors, a single-agent MET-TKI, rather than the currently recognized treatment regimen of EGFR-TKIs combined with MET-TKIs, might be sufficient to control the growth [45]. 

Notably, *MET* amplification is also one of the most frequent off-target mechanisms of acquired resistance to inhibitors of the ALK, ROS1, RET and TRK kinases as well as the KRAS GTPase, thus justifying the usage of combinations with MET-TKIs also in these settings [11,44].

In a small group of Asian NSCLC patients (n = 14) with acquired *MET* amplification after EGFR-TKI therapy, Crizotinib both in monotherapy and in combination with an EGFR-TKI provided promising outcomes with median PFS (mPFS) of 6.0 and 12.6 months, respectively [46]. Yet, response to monotherapy with Crizotinib may be more heterogenous, as suggested by another study comprising eight *EGFR*m+ NSCLC patients, who acquired *MET* amplification during EGFR-TKI treatment and exhibited mPFS of only 1.4 months [31]. A similar transient response was also observed in another case treated initially with Crizotinib followed by a brief period with Crizotinib in combination with Osimertinib [47]. In a larger group of 70 patients with acquired *MET* amplification after EGFR-TKI therapy, inhibition of both EGFR and MET seemed to be a more effective therapeutic strategy [48]. In that study, patients who received EGFR-TKI + Crizotinib experienced significantly longer PFS than those who received Crizotinib alone or chemotherapy (5.0 vs. 2.3 vs. 2.9 months, *p* = 0.010), however without OS being significantly different (10.0 vs. 4.1 vs. 8.5 months, *p* = 0.088). Another report provided clinical evidence for the efficacy of a combination regimen with either first- or third-generation EGFR-TKI together with Crizotinib after the emergence of *MET* amplification-mediated resistance to EGFR-TKIs [49]. One of the longest reported PFS for dual treatment with second-line Osimertinib and Crizotinib for acquired *MET* amplification was 19 months [50]. However, even in heavily pre-treated patients, the combination of EGFR-TKI and Crizotinib given as a sixth- and fifth-line treatment provided a clinical and radiographic response of about six and four months, respectively [51,52]. Combining Osimertinib and Crizotinib along with local ablative therapy due to oligoprogressive disease was also reported as a feasible treatment showing a PFS of approximately nine months [53]. Single cases of *EGFR*m+ NSCLC with acquired *MET* amplification detected by liquid biopsy of plasma cfDNA and treated with combined Osimertinib–Crizotinib have been reported with a PFS between three and four months [30,54]. 

In another case of acquired *MET* amplification detected by analysis of plasma cfDNA, the response to the combination of Crizotinib and Erlotinib was reportedly nine weeks due to rapid emergence of other resistance mechanisms [55]. Finally, a limited response of Afatinib combined with Crizotinib for acquired low-level *MET* amplification was also reported in a NSCLC patient harboring two synchronous uncommon *EGFR* mutations (exon 18 p.G719S and exon 19 p.L747S) and exhibiting sarcoma-like (spindle and/or giant cell) features [56]. 

As displayed in Table 1, targeting acquired *MET* dysregulation has been reported to have a longer effect on PFS when using the combination of EGFR-TKI + Crizotinib, rather than Crizotinib alone or chemotherapy, as the next line treatment upon progression on EGFR-TKIs. This may be explained by the fact that *EGFR*m+ NSCLCs progressing on first-line EGFR-TKI therapy usually remain dependent on EGFR-signaling, so that combining MET inhibition with continued EGFR-TKI treatment is more likely to be more effective than switching from EGFR to MET inhibition alone [43,57].

As presented in our cases, we recommend performing tumor rebiopsies in patients progressing on EGFR-TKIs, if feasible, to enable the next treatment based on a biomarker-matched approach. Indeed, recently published data support this approach by showing improved survival of *EGFR*m+ NSCLC patients progressing on first-line Osimertinib, when their second-line treatment was adjusted based on identified mechanisms of resistance at progression using tissue-based genomic analysis [58].

In all our five cases, tumor rebiopsies were taken at progression and tested for MET overexpression by IHC and for *MET* amplification by FISH and NGS, as previously described [32,59], since this is the procedure we also use in routine diagnostics. The rationale behind this approach is the fact that in most NSCLC cases with *MET* amplification this gene alteration results in overexpression, auto-aggregation, and ligand-independent activation of the MET receptor protein [20,32,44]. Thus, IHC-assessed MET expression may be used to screen for *MET* amplification, which subsequently needs to be verified by FISH/NGS. It may also be utilized to verify that in tumor cells with *MET* amplification, the encoded MET protein is overexpressed, as this is ultimately the target for MET-TKI treatment. Conversely, IHC-detected MET overexpression does not necessarily mean that the *MET* gene is amplified [20,32,57]. Indeed, there can be discrepancies between the results of MET overexpression obtained by MET-IHC and *MET* amplification assessed by FISH or by NGS. Moreover, there is no complete concordance between *MET* amplification detected by FISH and by NGS [22,43,44,57]. In addition to *MET* gene amplification or mutation, MET overexpression in NSCLC and other cancer types may also be caused by transcriptional/post-transcriptional/post-translational mechanisms [12,32,57,59,60,61]. Consequently, the incidence of MET overexpression in NSCLC, reportedly occurring in 15% to 75% of cases, is higher than that of *MET* amplification [6,11,12,60,61,62].

It is unclear whether cases of *EGFR*m+ NSCLC with IHC-assessed MET overexpression not linked to *MET* amplification or mutation are resistant to EGFR-TKIs. In this situation, MET upregulation is not an optimal predictor of response to MET-TKIs either, as these drugs appear less effective in NSCLC patients with MET overexpression in the absence of *MET* mutation or amplification [43,57]. One possible explanation is that IHC-determined MET expression in NSCLC does not necessarily reflect activation of MET signaling and tumor MET dependence [57]. Thus, evaluation of MET status by IHC alone remains a heterogeneous and debated predictor of response to TKIs, especially MET-TKIs [63]. This might also be due to the lack of standardized methods for performing MET-IHC in clinical studies, including the usage of different commercial antibodies against different MET epitopes and with variable sensitivity/specificity, as well as different scoring methods for evaluating MET expression levels [11,57,62,64]. In this respect, the automatized IHC procedure with the SP44 anti-MET antibody (by Ventana Medical Systems, Inc., Roche Diagnostics A/S, Hvidovre, Denmark) that we used for our five reported cases is the one routinely used for predictive diagnostics in most pathology departments and in most clinical trials employing MET-TKIs for *EGFR*m+ NSCLC patients with acquired *MET* amplification/overexpression [22,62]. 

Even though IHC-assessed MET overexpression may be utilized as a surrogate marker to screen for *MET* amplification, clinical studies have not entirely clarified the concordance between MET overexpression and *MET* mutation/amplification as predictive biomarkers and indicators of NSCLC dependence on MET signaling [57,58,60,64,65]. Because of that, the direct assessment of increased *MET*-GCN/*MET* amplification alone or combined with IHC-assessed MET overexpression is currently preferred for predicting responses to TKIs and rating MET addiction of tumors [43,57]. 

Nonetheless, IHC showed MET overexpression correlating with FISH-detected *MET* amplification in all our five cases. Moreover, strong concordance and comparable ORR were observed between MET-IHC positivity and MET-FISH positivity in the TATTON trial combining Osimertinib with Savolitinib for treating *EGFR*m+ patients with acquired *MET* amplification/overexpression [22]. These results support the continued use of IHC as a screening method that can complement FISH (and NGS) in selecting patients for combined treatment with Osimertinib and a MET-TKI. 

In any case, in keeping with our current data, FISH, despite being relatively laborious and observer-dependent [62], remains the most reliable method for detecting *MET* amplification in clinical NSCLC tissue samples with high sensitivity, minimal false negative rate, and the possibility of distinguishing *MET*-GCNG due to focal amplification of the *MET* genomic region on chromosome 7 from GCNG due to polysomy. Thereby, FISH-determined *MET* amplification remains the optimal biomarker to identify suitable candidates for MET-TKI therapy [62,63,64]. 

In comparison, amplification identified by NGS does not seem as robust as a predictive biomarker [66]. The reasons for that may be: (1) the high rate of false negatives by targeted NGS techniques in identifying *MET*-GCNG due to duplication of the whole chromosome 7 or parts of it larger than the *MET* region; (2) the subclonal nature of *MET*-amplified tumor cells in NSCLC, which can be captured by direct morphological assessment using FISH but may be “diluted” by DNA of other cells. Consequently, the subclonal MET amplification may be below the detection level in the bulky genomic DNA analyzed by NGS, which does not allow any morphological correlation [62,67]. Similar difficulties can be encountered using quantitative polymerase chain reaction (qPCR) or quantitative real-time-PCR (qRT-PCR) as methods to detect *MET* amplification in clinical settings [54,64]. Accordingly, the detection of *MET* amplification and its various levels by NGS (or qRT-PCR) analysis on cfDNA may be particularly challenging and result in a high rate of false negatives, as it is dependent on the level of obtained ctDNA [22]. In this respect, digital-droplet (dd)PCR has been proposed as an alternative, very sensitive method to detect *MET*-GCNG in both tissues and peripheral blood samples, which could be worth future clinical consideration, not least for cases where FISH is not applicable, given the very high concordance with FISH in detecting *MET* amplification [54,68,69,70]. 

In our cases 1, 3, and 4, NGS failed to detect the acquired *MET* amplification, which was, however, identified by FISH. Moreover, in case 2 NGS did not detect the low-level *MET* amplification revealed by FISH at first progression on Osimertinib (it did detect, though, the high-level *MET* amplification at progression on combined Osimertinib and Crizotinib treatment in this patient), whereas in case 5, NGS detected a *MET*-GCNG of 5 instead of the *MET*-GCN >8 uncovered by FISH. Our data are therefore consistent with other real-world studies showing that NGS can identify cases with high-level *MET* amplification (samples with GCN ≥10) and those that are also FISH-negative, but is not reliable for assessing the various levels of *MET* amplification, nor for distinguishing *MET*-GCNG due to true gene amplification from that caused by polysomy [44,62,67]. Our data also support the recent results by Hartmaier et al. in the TATTON trial with Osimertinib and Savolitinib to overcome MET-mediated resistance to EGFR-TKIs, which showed significantly higher concordance between IHC and FISH in detecting *MET* amplification than between NGS and FISH [22]. Furthermore, the levels of *MET* amplification and overexpression assessed by FISH and IHC, respectively, have been shown to bear a very important clinical impact in the SAVANNAH study [69,70]. Indeed, according to interim results from this trial, the efficacy of combined Osimertinib and Savolitinib in the subgroup of patients with a high-level *MET* amplification (defined by the authors as *MET*-GCN ≥10 by FISH with Vysis MET FISH Probe Kit, Abbott Molecular, Inc.) and MET overexpression (defined as IHC 3+ in ≥90% of tumor cells with the MET SP44 RxDx Assay by Ventana Medical Systems, Inc.) was improved in terms of ORR 49% and mPFS 7.1 months, as compared with the overall population (with IHC 3+ in ≥50% and/or *MET*-GCN ≥5 by FISH) showing an ORR of 32% and mPFS 5.3 months. These findings were also the reason for redefining the inclusion criteria for enriching the patient population most likely to derive clinical benefit in the current version of the study (NCT03778229).

Thus, despite the successful implementation of targeted NGS panels in routine clinical diagnostics of NSCLC [71], this method is still affected by challenges with respect to the detection of *MET* amplification and prediction of response to TKI treatment. As elegantly reviewed elsewhere, the most relevant of these challenges with NGS are the lack of consensus on cut-off values for detecting *MET* amplification; the overall amount, quality, and composition of tumor material (risk of insufficient tumor cell content and purity for sensitive detection of *MET* amplification); tumor heterogeneity; difficult distinction of *MET*-GCNG caused by focal gene amplification from that due to polysomy; difficult quantification of tumor clones with different amplification levels as compared to FISH, which results in poor concordance between NGS and FISH detection (discrepancy with FISH in different studies ranging between 37% and 75%); and NGS often unable to detect *MET* amplifications otherwise revealed by FISH [62,64,67]. 

Although FISH is currently the preferred method for determining *MET* amplification in NSCLC in routine clinical practice, it is not sufficiently standardized to allow ideal comparisons of different studies [44,62,64]. Especially, the *MET*-GCNG required to induce clinically significant MET overexpression and MET-signaling deregulation as well as the threshold for which each anti-MET treatment has an effect remain uncertain [43,44,57,71]. This reflects the subdivision in low- and high-level *MET* amplification in certain clinical studies, while others also include an intermediate-level, and even a top-level amplification, based on various *MET/centromere 7(CEN7)* ratios and/or average *MET*-GCN/cell that slightly differ from study to study [20,22,23,32,43,57,62,63,64,65,71,72,73,74]. In this respect, the *MET/CEN7* ratio is considered by many as a parameter reflecting true gene amplification, with a ratio ≥2 used for defining amplification in general, but also utilized by several studies to classify high-level amplification, whereas others have referred to a ratio of 1.8≤ *MET/CEN7* ≤2.2 for low-level amplification, 2.2< *MET/CEN7* <5 for intermediate-level amplification, and *MET/CEN7* ≥5 for high-level amplification. Instead, increased *MET*-GCN/cell can be determined either by true amplification of the gene (and possibly of the nearby chromosomal region) or by polysomy [62,63,64,72].

All five of our patients progressing on Osimertinib carried, as the most frequent co-alteration accompanying *MET* amplification, a *TP53* mutation. In two of these patients, this mutation was already present as a de novo alteration at baseline (cases 3 and 5, both smokers). This is consistent with the high rate of concomitant *TP53* mutations in *MET* amplified NSCLCs [11,12] and with our previous findings showing that up to 60% of *EGFR*m+ NSCLC patients with *MET* amplification and/or MET overexpression also harbored a *TP53* mutation [32]. These data may suggest a potential growth advantage for NSCLC cells with co-existing disruption of EGFR-, MET-, and p53-dependent signaling pathways. Indeed, it is known that *MET* amplification promotes proliferation and survival of TKI-treated *EGFR*m+ NSCLC cells by co-activating MAPK and PI3K/AKT signaling and by inhibiting the proapoptotic proteins BIM and APAF-1 [75,76,77]. Thus, additional proliferative and survival advantages may be provided to *MET* amplified tumor cells by concomitant disruption of the p53 tumor-suppressive function, which relies on p53-mediated cell-cycle arrest or apoptosis in response to not only DNA damage and hypoxia, but also mitogenic oncogenes (such as mutant *EGFR* or amplified *MET*) [78,79,80,81].

In the clinical setting, cases with acquired resistance to EGFR-TKIs linked to *MET* amplification are likely due to clonal selection of preexisting *MET* amplified cells during TKI treatment [6,11,57,77,82]. In support of this conception is the above-mentioned subclonal nature of *MET* amplification, which is observable by FISH and may be missed by NGS analysis [22,43,57], as also shown in our cases. Indeed, rare *MET* amplified cell subpopulations, representing <1% of cells in a tumor, have reportedly been uncovered in treatment-naïve cases of *EGFR*m+ NSCLC that subsequently progressed on EGFR-TKIs with *MET* amplification as the main mechanism of resistance [77,83]. Collectively, these observations suggest that dominant *MET* amplified clones may emerge from very low-frequent pre-existing cells under the selective pressure imposed by EGFR-TKIs.

Although the role of MET-mediated acquired TKI resistance is well established, the potential role of *MET* alterations in intrinsic TKI resistance (temporally defined as resistance causing tumor progression within three months from the treatment initiation [6,84]) is less clear [6]. Concurrent de novo *MET* amplification may be found in 2–8% of *EGFR*m+ NSCLC patients prior to EGFR-TKI therapy and may represent a potential mechanism of intrinsic resistance to EGFR-TKIs [6,20,32,66,85,86,87]. Indeed, these patients are prone to develop early progression on EGFR-TKI and are thought to obtain less benefit from a combined EGFR- and MET-TKI treatment than *EGFR*m+ patients with acquired resistance due to *MET* amplification during therapy [32,66,85,87]. However, some of these cases may respond to the EGFR-TKI + Crizotinib combination [6,88]. Moreover, a dramatic response to combined Erlotinib and Crizotinib was reported in a patient with *EGFR*m+ NSCLC harboring a very high level of de novo *MET* amplification with an *MET/CEN7* ratio >15 [89]. Similarly, durable response to combined EGFR-TKI and Crizotinib for concurrent *EGFR*m+ and de novo *MET* amplification was also observed in patients with sarcomatoid phenotype of NSCLC [90] and in a patient with two primary pulmonary adenocarcinomas, one harboring an *EGFR* ex19del and the other a *MET* amplification [91]. 

Yet, in keeping with concurrent de novo *MET* amplification as a mechanism of primary resistance to EGFR-TKIs, a retrospective Japanese study identified *MET* amplification at baseline in 11 of 35 *EGFR*m+ LAC patients treated with Gefitinib and showed that it predicted a higher risk of tumor progression and death [92]. Similarly, a targeted NGS analysis of 200 untreated *EGFR*m+ NSCLCs showed that concomitant *MET* amplification at baseline correlated with a shorter time to progression on first-line EGFR-TKI with a HR of 3.7 [93]. The importance of MET signaling in intrinsic resistance to TKIs was also underlined by another Japanese cohort, in which overexpression of the MET ligand HGF was detected in 29% of *EGFR*m+ NSCLC patients not responding to first-generation EGFR-TKIs [94]. In this study, the overexpression of HGF was more frequently associated with intrinsic or acquired TKI resistance than the *EGFR* p.T790M mutation or *MET* amplification [94].

Taken together, the reported results indicate that co-activation of MET signaling at baseline in *EGFR*m+ NSCLC is an event that may cause intrinsic resistance to EGFR-TKIs, but at the same time, it may also represent a potential target for first-line combination therapy aimed at disabling the inherent resistance to EGFR inhibition. The results from the ongoing FLOWERS study (NCT05163249) dedicated to *EGFR*m+ NSCLC patients with de novo *MET* amplification or overexpression will be important for finding the best clinical approach for such patients [95].

Nonetheless, we previously reported cases in which *MET* alterations (mutation, amplification, and/or overexpression) already present at baseline may or may not respond to Erlotinib alone [6,32]. Similarly, in a cohort of one hundred and thirty-three *EGFR*m+ NSCLCs, four cases harboring de novo *MET* co-mutations yet responding to first-generation EGFR-TKIs were described [83].

These variations in response to EGFR-TKIs could be explained by the level of concomitant *MET* amplification at baseline. In this regard, co-alterations in other oncogenic drivers, such as *EGFR*, *ALK*, *ROS1*, *KRAS*, *BRAF*, *ERBB2*, and *RET*, have been reported more frequently in NSCLCs with low-/intermediate-level *MET* amplification than in cases with high-level amplification, suggesting that MET is the main driver in the latter tumors [6,72,73]. Yet, the cases with *EGFR*m+ NSCLCs with concomitant de novo high-level *MET* amplification at baseline [20,32,72,89,91,92] may suggest tumor polyclonality, including heterogeneous clones with either mutated *EGFR* or amplified *MET* as a driver [6]. Supporting this notion, in a cohort of 200 consecutive patients with treatment-naïve metastatic *EGFR*m+, FISH analysis revealed concomitant *MET*-GCNG at baseline in 52 of them, which was due to polysomy in 46 patients and true *MET* amplification (as *MET/CEN7* >2) in the other six [73]. Notably, the level of *MET*-GCNG displayed by the 46 patients with polysomy did not correlate with the response to EGFR-TKIs, while five out of the six patients with a de novo true *MET* amplification displayed considerably worse response to these drugs, with the two cases exhibiting the highest *MET/CEN7* ratio progressing already within the first month of treatment [74]. Thus, patients with *EGFR*m+ NSCLC with true high-level *MET* amplification at baseline may harbor clones of MET-driven tumor cells that may very rapidly take over when EGFR signaling is inhibited and may cause intrinsic resistance to EGFR-TKIs, resulting in very rapid cancer progression [6,73].

Hence, NSCLC cells with de novo deregulated MET signaling may already be present at baseline; however, their clinical significance in intrinsic resistance seems heterogeneous, possibly because of the polyclonality of resistance mechanisms and different levels of MET signaling deregulation or tumor cell addiction to this signaling. Accordingly, the role of these cells in intrinsic TKI resistance requires further investigation in larger cohorts. These studies may also elucidate to which extent the above-mentioned discrepancies regarding response to TKIs in *EGFR*m+ cases with de novo co-amplification of *MET* are imputable to the lack of standardized methods for determining *MET* amplification. 

While we are expecting conclusive data from the clinical trials currently investigating new MET inhibitors in the setting of acquired resistance to EGFR-TKIs in [11,12,67,96], *EGFR*m+ NSCLC patients with both de novo and acquired *MET* amplification, who cannot be included in these trials, may be offered a combination of EGFR-TKI and Crizotinib, which is a feasible and reasonable approach. Notably, the longest PFS in the real-world cases reported so far (18 and 19 months, respectively, in our case 4 and report 7 in Table 1) was observed in the absence of multiple genomic and/or phenotypic co-alterations. The ongoing studies with more potent MET inhibitors also try to answer the question of which EGFR-TKI generation should be preferred in combination treatment. In this respect, *EGFR*m+ NSCLC patients with *MET* amplification have probably a minor propensity to metastasize to the central nervous system (CNS); thus, combinations of MET inhibitors with EGFR-TKIs that are less CNS penetrable than Osimertinib are being investigated too [11,96].

## 4. Materials and Methods

### 4.1. Literature Searches

Available literature on *MET* amplification as both de novo and acquired mechanisms of resistance to EGFR-TKI treated with Crizotinib was searched in the following databases: PubMed, Cochrane, and Medline.

### 4.2. Immunohistochemistry (IHC) for MET Receptor Protein 

The immunostaining for membranous and cytoplasmic expression of MET receptor was performed as previously described [32,59]. Briefly, 2.5 μm thick formalin-fixed paraffin-embedded (FFPE) tissue sections from each sample were stained using a Roche Ventana BenchMark ULTRA automated slide immunostainer (Ventana Medical Systems Inc.; Roche Diagnostics A/S, Hvidovre, Denmark), Ultra Cell Conditioning solution (CC1) pretreatment for 8 min at 95 °C, four CC1 treatments (20, 36, 52, and 64 min), and incubation with pre-diluted CONFIRM anti-MET (clone ID, SP44) rabbit monoclonal antibody (mAb) (Ventana Medical Systems, Inc.; Roche Diagnostics A/S, Hvidovre, Denmark) for 16 min. The immune reactions were visualized using an ultraView DAB Detection Kit (Ventana Medical Systems, Inc.; Roche Diagnostics A/S, Hvidovre, Denmark) and hematoxylin counterstaining (Ventana Medical Systems, Inc.; Roche Diagnostics A/S, Hvidovre, Denmark), following the manufacturer’s recommendations. 

MET protein expression was scored in a blinded manner (before knowing the FISH results) by one observer (E.S.-R.), assessing staining intensity (negative, weak, moderate, or strong) and the percentage of stained cells, thereby defining four diagnostic “immunoscores”, *i.e.*, 3+ (strong intensity in ≥50% of cells), 2+ (moderate intensity in ≥50% of cells), 1+ (weak intensity in ≥50% of tumor cells), and 0 (no staining or <50% of tumor cells stained). Both 2+ and 3+ are considered as indicative of MET upregulation (“MET-positive”) as opposed to no upregulation (“MET-negative”), as previously described [32,33]. Endothelial cells or bronchial/alveolar epithelial cells present in the tissue sections were used as internal controls, since they can display weak and weak-moderate intensity of MET expression, respectively, as previously reported [32,33]. Image acquisition was obtained by digital scanning of the slides with a Nano Zoomer S210 slide scanner (Hamamatsu, Ballerup, Denmark) and the digital slide viewing software Sectra Workstation IDS7, v.24.1.15.5568 (Sectra AB, Linköping, Sweden).

### 4.3. Fluorescence In-Situ Hybridization (FISH) for MET Amplification

FISH was performed with the Zyto-Light SPEC MET/CEN7 dual-color probe (Zytovision GmbH, AH diagnostics A/S, Tilst, Denmark) that detects the *MET* gene and the centromeric portion of the *MET*-harboring chromosome 7 (*CEN7*), as previously described [32,33]. Briefly, slides were scanned using a ×63 objective and appropriate filter sets (automated upright Leica DM4 B fluorescent microscope; Leica Microsystems, Brønshøj, Denmark), using normal fibroblasts, leukocytes, and endothelial cells as internal controls, and individually analyzing 100 tumor cell nuclei (20 neighboring tumor cell nuclei from 5 random areas of homogenous distribution of *MET* signals) with the ×100 objective, counting *MET* (green) and *CEN7* (orange) signals. Representative images were acquired using a 19 mm sCMOS Leica DFC9000 camera incorporated with the microscope after identification of representative areas with the Leica LAS X Navigator Software, v.3.6.0 Widefield (Leica Microsystems, Brønshøj, Denmark). FISH was assessed by two readers (E.S.-R. and a trained and experienced laboratory technician). The tumor samples were classified into the following four groups of *MET* amplification status [32,33]: (A) High-level *MET* gene copy number gain/gene amplification (GCNG/GA) = *MET*/centromere 7 (*CEN7*) ratio ≥2.0 or an average *MET*-GCN/cell ≥6.0 or ≥10% of tumor cells with ≥15 *MET* signals (“clusters”); (B) Intermediate-level *MET*-GCNG/GA =≥50% of tumor cells with ≥5 *MET* signals; (C) Low-level *MET*-GCNG/GA =≥40% of tumor cells with ≥4 *MET* signals; and (D) No *MET*-GCNG/GA = none of the above criteria fulfilled.

### 4.4. Analysis of Therapeutic Targets and TKI Resistance Mechanisms in Tumor Biopsies and Liquid Biopsies

To identify TKI-resistance mechanisms during treatment, baseline biopsies and longitudinal rebiopsies from new consecutive metastatic lesions emerging during tumor progression were analyzed histologically and by IHC with specific markers (CK7, CK5, TTF1, p40, synaptophysin, chromogranin, CD56, E-cadherin, and vimentin) for possible phenotypic transformation to small-cell carcinoma or squamous carcinoma and for epithelial-mesenchymal transition (EMT), as previously described [97,98]. Immunohistochemical expression of MET receptor and FISH for *MET* amplification were assessed as described above.

For targeted next-generation sequencing (NGS) analysis, genomic DNA was purified using the Maxwell RSC Blood DNA kit (Promega, Madison, WI, USA) for cytological samples and an in-house crude DNA extraction method for FFPE material (protocol available upon request). The genomic DNA was quantified using the Qubit™ dsDNA HS Assay on a Qubit™ 4 Fluorometer (Thermo Fisher Scientific, Roskilde, Denmark). At the time of diagnosis, the mutational status of *EGFR* and 21 other lung cancer-relevant genes was determined using the AmpliSeq Colon and Lung Research Panel v.2 on the Genexus™ system (Thermo Fisher Scientific, Roskilde, Denmark). Rebiopsies were investigated for single nucleotide variants (SNVs), short indels, and copy number variations (CNVs) across 161 unique cancer-associated genes using the Oncomine Comprehensive Assay v.3 according to the manufacturer’s instructions (Thermo Fisher Scientific, Roskilde, Denmark), as previously reported [98,99]. After the preparation of amplicon-based libraries, the DNA was sequenced on the Ion Torrent™ GeneStudio™ S5 Plus System (Thermo Fisher Scientific, Roskilde, Denmark) according to the manufacturer’s instructions. 

RNA was purified from diagnostic biopsies and tumor rebiopsies utilizing the Maxwell RSC instrument (Promega, Madison, WI, USA) with a Maxwell RSC RNA FFPE kit (Promega) and quantified with the Qubit™ RNA HS Assay. NGS analysis of RNA from these specimens was performed to identify gene fusions causing primary or acquired EGFR-TKI resistance using the Archer FusionPlex Lung kit, which analyses 14 gene fusions according to the manufacturer’s instructions (ArcherDX Inc., Boulder, CO, USA), as reported [99].

Additionally, liquid biopsies of cfDNA from 3.5 mL of plasma were analyzed at baseline when EGFR-TKI treatment was started, after two months of treatment, and at progression for relevant DNA mutations. Plasma was isolated and cfDNA was purified with the Cobas cfDNA Sample Preparation Kit (Roche Diagnostics, Mannheim, Germany) and quantified with the Qubit™ dsDNA HS Assay, as described above. NGS analysis of cfDNA was performed using the Oncomine Lung cfDNA NGS-assay, which analyzes hotspot mutations in 11 genes, according to the assay’s instructions (Thermo Fisher Scientific, Roskilde, Denmark), as previously described [98,99].

All NGS data were analyzed using the Torrent Browser (v.5.14.0) and the Ion Reporter (v.5.14) software (Thermo Fisher Scientific, Roskilde, Denmark). Variants were visualized by the Integrative Genomics Viewer (https://igv.org) [100], classified according to ACMG classification [101], and further analyzed using the OncoKB (https://www.oncokb.org), COSMIC (https://cancer.sanger.ac.uk/cosmic), and ClinVar (https://www.ncbi.nlm.nih.gov/clinvar) databases [102,103,104].

## 5. Conclusions

Based on real-world data, the combination of EGFR-TKI and Crizotinib in patients with *EGFR*m+ NSCLC progressing on EGFR-TKIs due to acquired *MET* amplification is feasible and currently a reasonable option while we are awaiting the results of clinical trials with new, more potent MET inhibitors. 

*EGFR*m+ NSCLC patients with high-level *MET* amplification received the highest benefit from Crizotinib compared to less *MET* amplified cases. 

Furthermore, the response to combined EGFR-TKI and Crizotinib is longer in cases without multiple co-existing genomic or phenotypical alterations. 

Given the heterogeneity of *MET* amplification, rebiopsies should be examined with all three methods (MET-IHC, MET-FISH, and MET-NGS) for thoroughly defining the level of amplification and co-existing alterations. 

*EGFR*m+ NSCLC with de novo *MET* amplification, an uncommon but clinically relevant molecular configuration, also deserves special diagnostic awareness and therapeutic consideration, as combined EGFR-TKI/MET-TKI treatment will be needed up-front in this setting.

## Figures and Tables

**Table 1:** Overview of clinical efficacy of combination treatments with Crizotinib and EGFR-TKIs for acquired *MET* amplification in EGFRm+ NSCLC patients. * Only genomic or phenotypical alterations considered as potential mechanisms of TKI resistance are indicated.

	The Preceding Treatment	Detection Methods	Number of Patients	Treatment for Acquired *MET* Amplification	PFS Months	Multiple Genomic/ Phenotypical Co-Alterations in Rebiopsy (Yes/No) *	References
1	Osimertinib (9 months)	NGS, IHC, and FISH (all cases)	1 (case 1)	Crizotinib + Osimertinib	9	Yes	current article
	Osimertinib (6 months)		1 (case 2)	Crizotinib + Osimertinib	6	Yes	
	Osimertinib + Alectinib (9 months)		1 (case 3)	Crizotinib + Osimertinib	9	Yes	
	Atezolizumab (4 months)		1 (case 4)	Crizotinib + Gefitinib	18	No	
	Osimertinib (26 months)		1 (case 5)	Crizotinib + Osimertinib	11	Yes	
2	1st gen. EGFR-TKI	FISH	14	Crizotinib (8)	6.0	No	[46]
				Crizotinib + EGFR-TKI (6)	12.6		
3	1st/2nd gen. EGFR-TKI as first line (8) chemotherapy (6) as second line	NGS FISH	8	Crizotinib (2) Crizotinib + EGFR-TKI (6)	1.4	Yes	[31]
4	3rd gen EGFR-TKI	FISH	1	Crizotinib alone	1.5	Yes	[47]
5	1st gen. EGFR-TKI (40) 2nd gen. EGFR-TKI (3) 3rd gen. EGFR-TKI (26) 1st gen. EGFR-TKI after 2. line chemotherapy (1)	NGS FISH	67	Crizotinib (10) Crizotinib + EGFR-TKI (35) Chemotherapy (22)	2.3 5.0 2.9	Yes	[48]
6	1st gen. EGFR-TKI (4) 2nd gen. EGFR-TKI (7)	NGS	11	1st/2nd gen. EGFR-TKI + Crizotinib (6) 3rd gen. EGFR-TKI + Crizotinib (5)	5.8	Yes	[49]
7	1st gen. EGFR-TKI Pemetrexed 3rd gen. EGFR-TKI	NGS FISH	1	Crizotinib + Osimertinib	19	No	[14]
8	Osimertinib rechallenge	NGS FISH	1	Crizotinib + Osimertinib	6	No	[15]
9	Chemotherapy	NGS FISH	1	Crizotinib + Erlotinib	4	Yes	[16]
10	Erlotinib Local ablative therapy	NGS FISH	1	Crizotinib + Osimertinib + local ablative therapy	9	Yes	[17]
11	Chemotherapy	cfDNA	1	Crizotinib + Osimertinib	4	Yes	[18]
12	Gefitinib	cfDNA	1	Crizotinib + Osimertinib	3	Yes	[19]
13	Erlotinib	cfDNA	1	Crizotinib + Erlotinib	2	Yes	[55]
14	EGFR-TKIs	NGS, IHC and FISH	1	Crizotinib + Osimertinib	11	Yes	[22]
15	Afatinib	NGS	1	Crizotinib + Afatinib	4	Yes	[56]

## Data Availability

The data presented in this study will be made available by the authors on request. The data are not publicly available due to institutional and national ethical and privacy restrictions regarding the included patients.
